# The increasing instance of negative emotion reduce the performance of emotion recognition

**DOI:** 10.3389/fnhum.2023.1180533

**Published:** 2023-10-13

**Authors:** Xiaomin Wang, Shaokai Zhao, Yu Pei, Zhiguo Luo, Liang Xie, Ye Yan, Erwei Yin

**Affiliations:** ^1^Academy of Medical Engineering and Translational Medicine, Tianjin University, Tianjin, China; ^2^Defense Innovation Institute, Academy of Military Sciences (AMS), Beijing, China

**Keywords:** affective computing, emotion recognition, EEG, ECG, experimental protocol designing, negative emotion

## Abstract

**Introduction:**

Emotion recognition plays a crucial role in affective computing. Recent studies have demonstrated that the fuzzy boundaries among negative emotions make recognition difficult. However, to the best of our knowledge, no formal study has been conducted thus far to explore the effects of increased negative emotion categories on emotion recognition.

**Methods:**

A dataset of three sessions containing consistent non-negative emotions and increased types of negative emotions was designed and built which consisted the electroencephalogram (EEG) and the electrocardiogram (ECG) recording of 45 participants.

**Results:**

The results revealed that as negative emotion categories increased, the recognition rates decreased by more than 9%. Further analysis depicted that the discriminative features gradually reduced with an increase in the negative emotion types, particularly in the θ, α, and β frequency bands.

**Discussion:**

This study provided new insight into the balance of emotion-inducing stimuli materials.

## 1. Introduction

Affective computing is aims to give machines the ability to interpret and simulate human emotional states (Tao and Tieniu, 2005). A significant advance in the last few decades can be attributed to the updates and developments in new feature extraction, machine learning algorithms, and portable devices (Arya et al., 2021). Advances in hardware have increased the emotional modality that machines can perceive, such as Electroencephalogram (EEG), Electrocardiography (ECG), Galvanic Skin Response (GSR), and Electromyography (EMG). Meanwhile, advances in software have helped machines mine the emotional component of multimodal data more effectively.

Achieving better emotion recognition performance is a constant topic for emotional human-computer interface (HCI) researchers. This led to many previous studies on affective HCI (aHCI) focusing on algorithm application. However, in Parkinson’s criticism of the “machine” end, such as novel feature extraction or the novel three areas of emotion research (individual, interpersonal, and representational components of emotion), “human” is the determining factor (Subramanian et al., 2016). Recently, some researchers have attempted to make some changes to bring human factors into emotional computing (Miranda-Correa et al., 2018; Song et al., 2019; Lee and Yoo, 2020). Particularly, negative emotion recognition has attracted much attention (Vaudable and Devillers, 2012; Gouizi et al., 2014).

Negative emotions are usually defined as these with low valences (Zheng et al., 2017). Negative emotions often lead to mental stress, as well as a decrease in attention and concentration. Therefore, it plays a significant role in the survival and adaptation of individuals (Vaskinn et al., 2017; Woodard et al., 2022). From this perspective, recognizing negative emotions is extremely important for affective HCI (Lee and Yoo, 2020). Research on negative emotions can help guide the diagnosis and treatment of mood disorders (Terhürne et al., 2022; Wu et al., 2022). Typical application scenarios for negative emotion detection include the online mental healthcare platforms or an indicator to assess the quality of call center conversations (Vaudable and Devillers, 2012; Gouizi et al., 2014; Dheeraj and Ramakrishnudu, 2021). However, recent studies have demonstrated that it is more difficult to accurately classify negative emotions than neutral and positive emotions (Calvo et al., 2009; Wang et al., 2020). For example, in SEED IV, the accuracies of EEG signal-based classifier in the case of neutral and positive emotions were 80 and 78%, respectively, whereas those for sadness and fear were 63 and 65% (Zheng et al., 2018). Zhuang et al. (2018) investigated the classification accuracy of six discrete emotions, including four negative emotions. The accuracy of the latter four emotions was approximately 50%, whereas the accuracies of neutral and positive emotions reached 70 and 62%, respectively. In terms of EEG signal characteristics, negative emotional states exhibited higher energy in the delta, theta, and alpha frequency bands compared to neutral emotions (Feradov and Ganchev, 2019). And some studies showed that high-arousal negative emotions are associated with increased physiologic reactivity such as elevated blood pressure (Gordon and Mendes, 2021).

Dozens of databases have been proposed for various purposes in affective computing research. They can be classified into three types based on their motivation. First, several studies have explored the representation of emotions in different physiological signal (Kim and André, 2008; Soleymani et al., 2011; Katsigiannis and Ramzan, 2017; Leelaarporn et al., 2021; Chen J. et al., 2022; Yu et al., 2022). For example, some researchers have attempted to induce negative emotions, such as disgust and amusement, by watching videos and synchronizing the acquisition of multiple physiological electrical signals (Soleymani et al., 2011). Their findings suggest that multimodal data can improve the accuracy of emotion recognition. Second, other databases were created to explore altering stimulus materials to increase the intensity of emotional arousal (Koelstra et al., 2011; Carvalho et al., 2012; Aydemir, 2017; Raheel et al., 2021; Elkobaisi et al., 2022; Kadiri and Alku, 2022). Typically, some researchers created immersive multimedia content for the audiovisual elicitation paradigm alone. Finally, other researchers have designed new experimental paradigms (Zheng and Lu, 2015; Samson et al., 2016; Subramanian et al., 2016; Menezes et al., 2017; Miranda-Correa et al., 2018; Sharma et al., 2019; Siddiqui et al., 2022).

For example, researchers designed an experiment in which subjects watched videos while simultaneously assessing their emotions and annotating their sentiment in real-time using a joystick (Sharma et al., 2019). This study provides an open-source real-time emotion-annotation database. In general, most databases aim at experimental paradigm innovation. However, to our knowledge, no database has formally explored the effects of increasing negative emotion types on subjects’ emotion evocation.

EEG is a bio signal that offers not only safety and non-invasiveness (Hu et al., 2022) but also a high temporal resolution, allowing for the recording of brain electrical activity at millisecond rates (Hua et al., 2022). Due to its capability to capture neural electrical activity, EEG is highly valuable in comprehending human cognitive processes, attention, emotional responses (Li et al., 2020; Schlumpf et al., 2022). ECG is capable of providing crucial insights into the heart’s functioning (Shi et al., 2022). Features extracted from ECG signals can be utilized to analyze variations in heart rate resulting from emotional changes (Chen P. et al., 2022; Rabbani and Khan, 2022). EEG and ECG were selected.

The major contributions of this study can be summarized as follows: (1) A new multimodal dataset of emotions called the continuous upgrading multi-model affect elicitation (CUMULATE) database was developed to assess the effects of increasing negative emotion types on emotion recognition. As far as we know, no similar application dataset has been proposed to data. (2) A fundamental reason for the difficulty in achieving good performance for negative emotion recognition was systematically described. We introduced a bioinformatics volcano plot to depict the changes in each dimension of emotional traits with the increase types in negative emotions. (3) Our results provide suggestions to avoid the effects of unbalanced negative emotion materials on emotion recognition.

The rest of this paper is organized as follows: The second part describes the main methods used in this research, including experimental design, multi-model data recording, and the feature extraction process. The experimental results section presents the results of the emotion classification based on EEG and ECG. The main reasons for the decrease in classification accuracy with an increase in negative emotion types were also analyzed. In the discussion section, we discussed biological mechanisms and suggestions. Finally, we presented the conclusions of this study.

## 2. Experimental setup

### 2.1. Experimental scenarios

**Figure 1:**
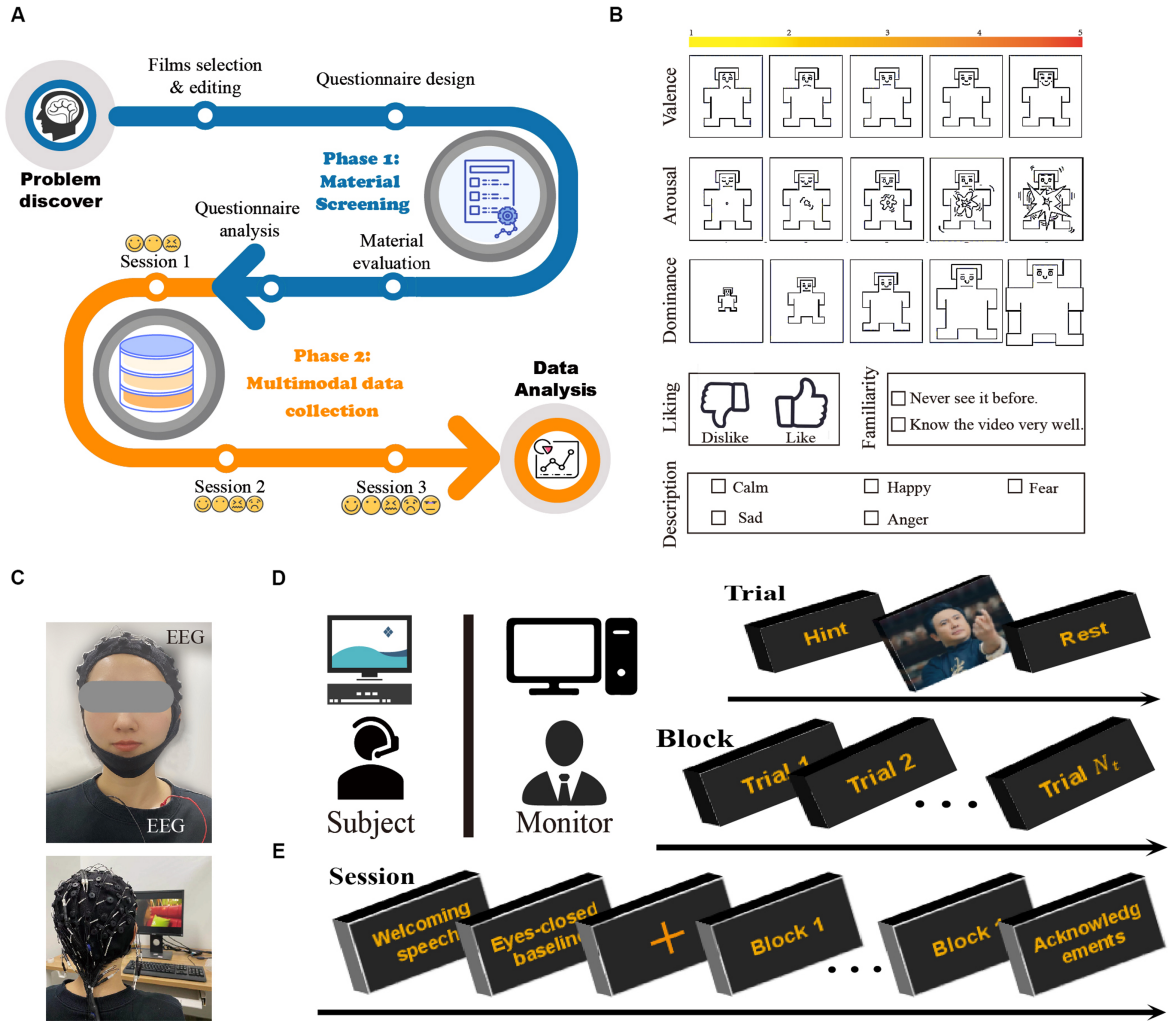
**(A)** Collection process of the dataset. CUMULATE study overview. **(B)** Images used for self-assessment. **(C)** Placement of physiological sensors. Sixty electrodes were used to record EEG and three for ECG. Experimental settings. **(D)** The experiments were performed in a separate laboratory environment. **(E)** Timeline for an experimental protocol.

The main objective of this work was to explore the effect of adding stimuli with negative valence to the paradigm on the accuracy of emotion classification. The entire process of the CUMULATE dataset was shown in Figure 1A. For this purpose, three sessions were designed in the study. In session 1, there were video clips of three types of emotions: calmness, fear, and happiness. Sadness was added in session 2, and anger was further included in session 3. In each session, the participants watched varying numbers of movie clips, and EEG and ECG data were recorded synchronously. All subjects learned the experimental process and equipment safety and signed the informed consent form before the experiment. The experiment was approved by the Ethics Committee of Tianjin University (TJUE-2021-138).

### 2.2. Stimulus selection

Two phases were included in the selection of stimulating materials. In the first phase, seven members of our research group constructed an initial database of 129 movie clips. In this process, according to EMDB’s requirements (Carvalho et al., 2012), the following three criteria were referenced: (a) stability of video content; (b) persisting characters in the scene, except the neutral material; and (c) positive and negative emotions not being elicited as coexisting. Each clip was edited according to acting scene and the emotional content throughout its duration to ensure consistency. Based on the rating results, 29 video clips were selected. In the second phase, 27 annotators (y: 26.26 ± 3.30, 8 females) were invited to watch these 29 video clips. During each video, the participants self-rated their emotions according to a self-assessment questionnaire as displayed in Figure 1B.

Both dimensional and discrete emotion models (Eerola and Vuoskoski, 2011) were included in the questionnaire. The dimensional emotion model involved five dimensions rated on a five-point Likert scale: valence (1 indicates extremely unpleasant, 5 indicates extremely pleasant), arousal (1 indicates calm, 5 indicates exciting), dominance (1 indicates submissive, 5 indicates sense of control) (Lu et al., 2005), liking, and familiarity. This study investigated five emotions in a discrete emotion model: happiness, sadness, fear, anger, and calmness. At the end of the second phase, the emotion label (emotion/rage + word/description) was determined for each movie clip.

**Figure 2:**
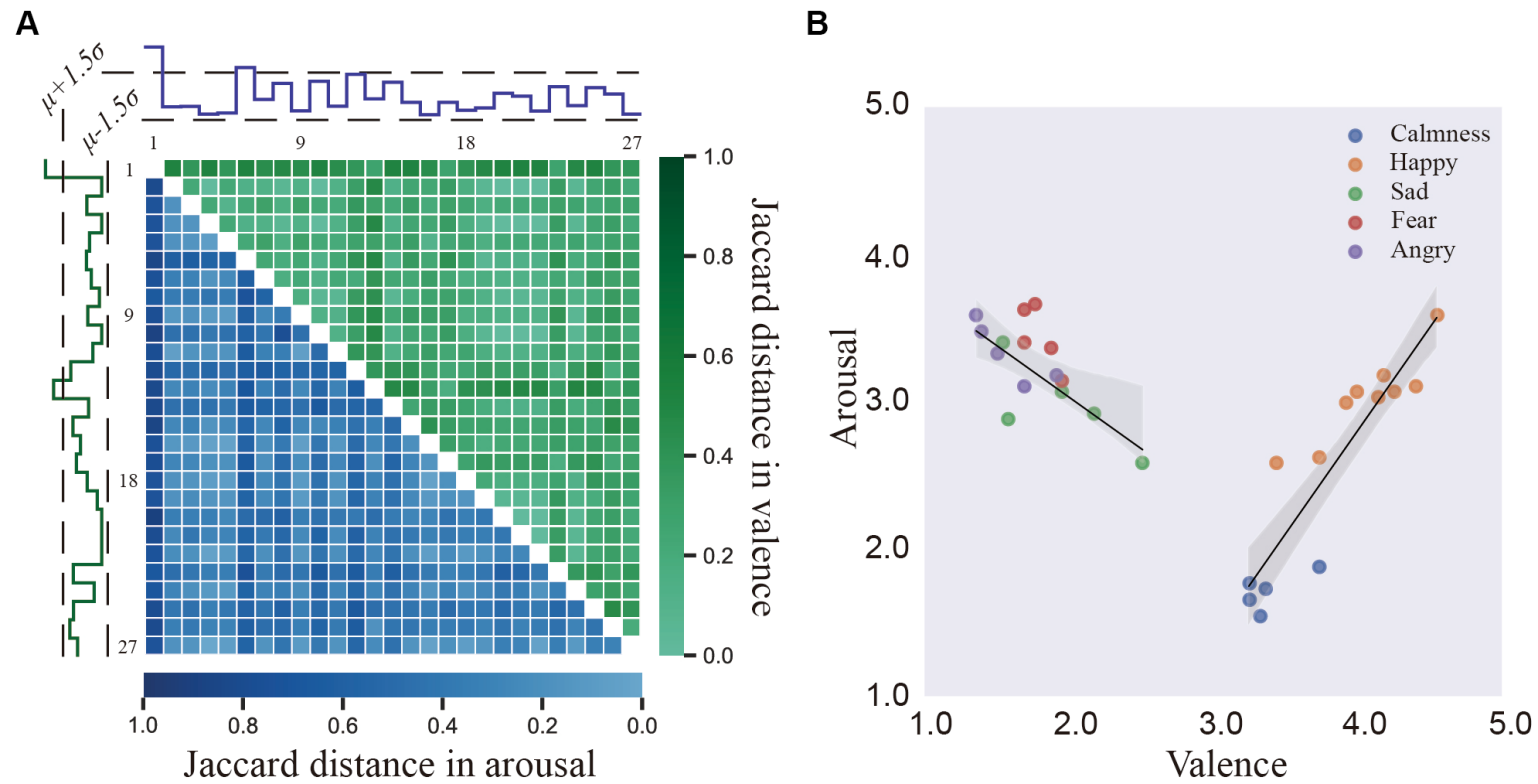
**(A)** The Jaccard distance between 27 annotators in valence and arousal. **(B)** The mean locations of the stimuli on the arousal–valence plane (AV plane).

According to DECAF (Abadi et al., 2015), to better estimate general affective perception, the outliers found in each video clip were discarded (Real and Vargas, 1996). According to the annotators’ rating, video clips were divided into high- and low-score sets along the valence (V) or arousal (A) axes with a 3 threshold. Then, the Jaccard distances between each pair of annotators for the high- and low-score sets were computed to form a distance matrix $D\left(\mathrm{i,j}\right)=1-\frac{\left|H_{i}\&H_{j}\right|}{\left|H_{i}\right|}$ , where 𝐻𝑖 is a binary vector denoting the high- or low-score of the i-th annotator’s rating and $\left|H_{i}\right|=29$ . $\left|H_{i}\&H_{j}\right|$ denotes the number of equal elements in corresponding index between $H_{i}$ and $H_{j}$ . Finally, the median absolute deviation (MAD) was calculated as the standard deviation of D′ upper- or lower- triangular. If the standard deviation of the i-th row of D was over 1.5 times of MAD, the corresponding annotator was considered outliers. As shown in Figure 2A, two annotators in V dimension and one annotator in A dimension was removed as outliers, respectively.

### 2.3. Stimulus assessment

Figure 2B presents the distributions of V/A ratings provided by the remaining 24 (27–3) annotators for the video clips. Colored dots denote five emotions with different V/A values: calmness (high valence and low arousal; HVLA, blue), happiness (HVHA, orange), sadness (LVHA, green), fear (LVHA, red), and anger (LVHA, purple). A U-shaped curve was observed, similar to (Abadi et al., 2015), implying the difficulty of evoking low arousal but a strong valence response (Lang et al., 2005; Koelstra et al., 2011). A linear regression model was adopted to check whether the valence ratings of the video stimuli were associated with arousal ratings. In the entire set of video stimuli, the linear correlation between arousal and high valence rating was substantial (*R*^2^ = 0.8307, *F* value = 58.8808, *p* value <0.001, b = 1.4183) compared with the correlation between arousal and low valence ratings (*R*^2^ = 0.4998, F value = 12.9901, *p* value <0.005, b = −0.7082). The shadow in Figure 2B is a 95% confidence interval for this regression model.

**Figure 3:**
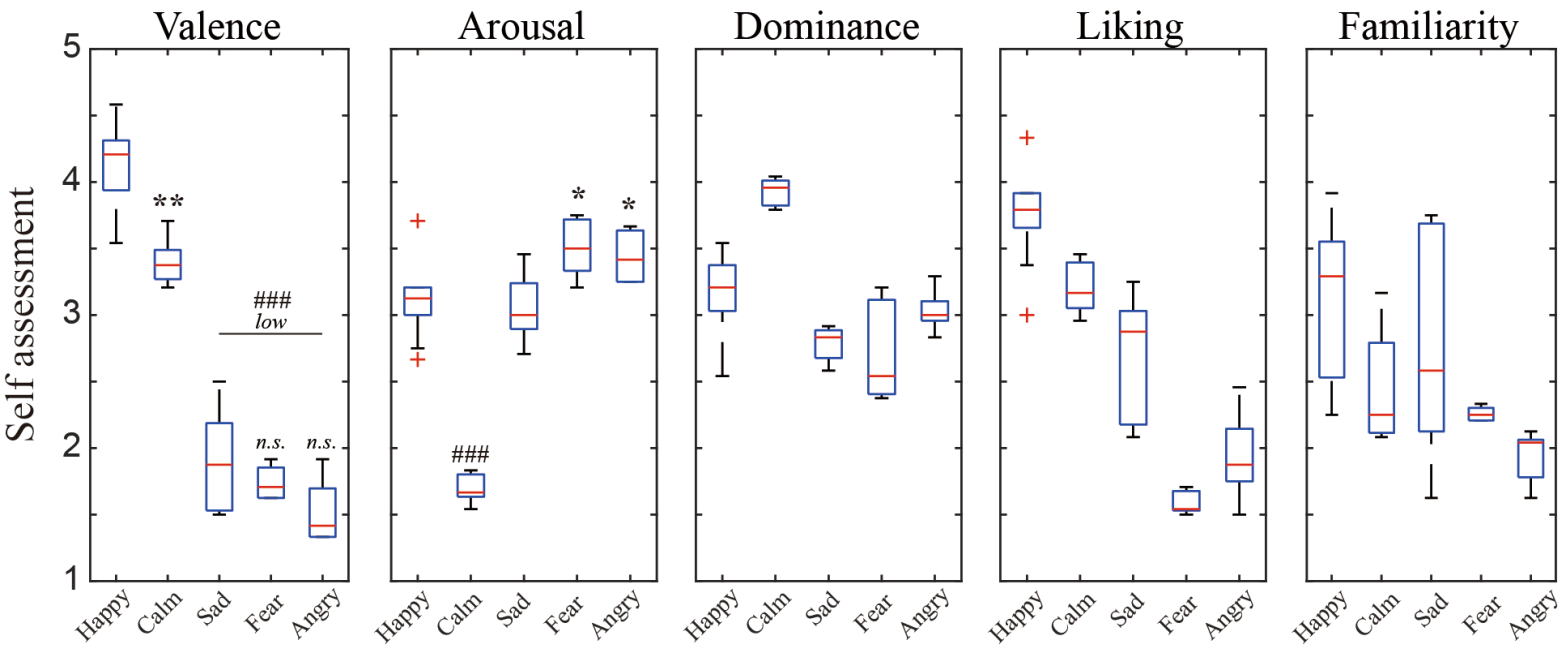
The distribution of the participants’ subjective ratings per scale for the five affects elicitation conditions.

Figure 3 presents the distribution of user ratings for the above-mentioned five discrete emotions in terms of arousal, valence, engagement, liking, and familiarity. Despite the inter-individual differences in scores, significant differences between the conditions in V/A scores reflected the successful elicitation of target emotional state (Koelstra et al., 2011).

**Figure 4:**
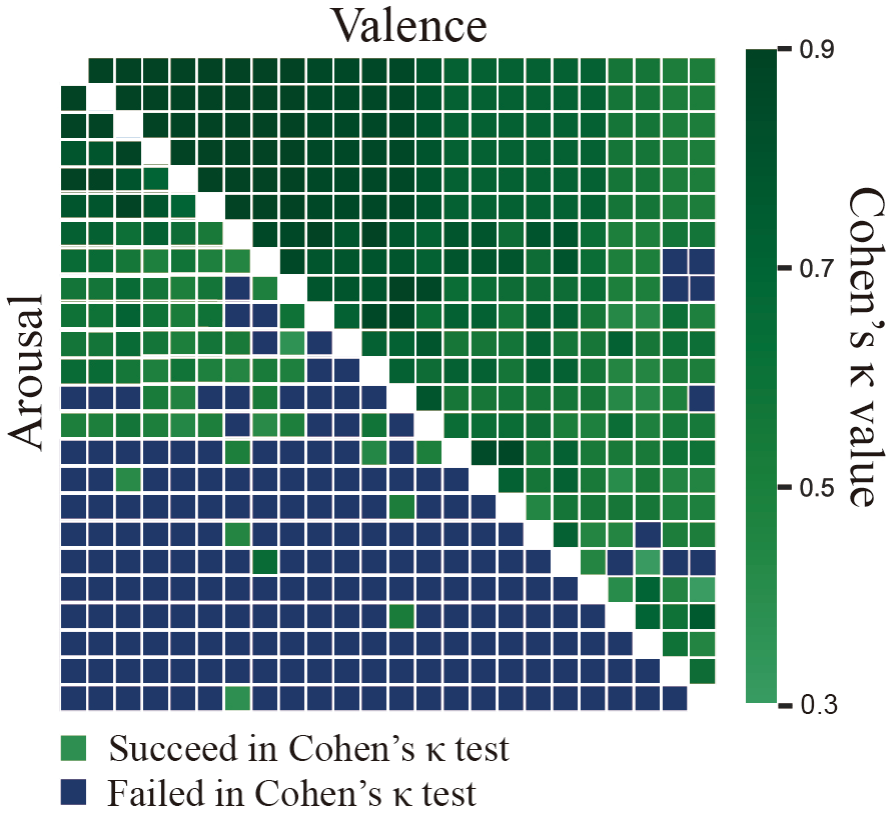
The agreement between the ground truth and the participant ratings using Cohen’s Kappa measure assumes that the ground-truth AV labels were provided by annotators.

The consistency of the V/A ratings among the annotators was evaluated using Krippendorff’s alpha metric, and they were found to be 0.69 and 0.30, respectively. This suggested that the annotators’ V/A scores for the mood and the arousal of the video clips were more consistent in our database than in ASCERTAIN with corresponding values of 0.58 and 0.12, respectively (Subramanian et al., 2016). Furthermore, as illustrated in Figure 4, Cohen’s Kappa test was adopted to explore the consistency of annotator pairs, and the results implied more consistent pairs in valence than in arousal. Overall, these measures showed that, while individual differences existed in the affective perception of video clips, there was substantial agreement among the assessments of each annotator, suggesting the effectiveness of the selected video clips for emotion elicitation.

### 2.4. Signal recording and synchronization

EEG was acquired using the NEUROSCAN acquisition system and a SynAmps RT 64-channel amplifier.^1^ Sixty electrodes were used to record EEG(FP1, FPZ, FP2, AF3, AF4, F7, F5, F3, F1, FZ, F2, F4, F6, F8, FT7, FC5, FC3, FC1, FCZ, FC2, FC4, FC6, FT8, T7, T5, C3, C1, CZ, C2, C4, T6, T8, TP7, CP5, CP3, CP1, CPZ, CP2, CP4, CP6, TP8, P7, P5, P3, P1, PZ, P2, P4, P6, P8, PO7, PO5, PO3, POZ, PO2, PO4, PO6, O1, OZ, O2). The ECG was acquired using BIOPAC MP150 acquisition system and ECG100C model.^2^ Three electrodes for ECG (RA, LA, LL). They were synchronized *via* a common parallel bus, with an accuracy of approximately 1 ms.

### 2.5. Experiment protocol

A total of 45 subjects (y: 26.20 ± 4.74, 10 females) participated in the experiment, with 15 subjects in each session. The experiments were conducted in an isolated laboratory under controlled light, as displayed in Figure 1D. Before the start of the experiment, the subjects were given verbal and written instructions regarding the experiment. After all the questions about the experiment were addressed and all issues were clarified, the subjects were asked to sign an informed consent form. Physiological sensors were then attached to the participants, who sat 60 cm (Wang et al., 2018) from a 15-inch screen (Hettich et al., 2016), as displayed in Figure 1C. The experimental process is illustrated in Figure 1E. Each trail contained progress and hints, video clips playing and emotional recovery. Before each video was played, the type of video determined from the statistical results of the user self-evaluation questionnaire was displayed promptly (Dutton and Aron, 1974; Satpute et al., 2016). The subjects controlled their progress independently by pressing the space button on each link. At the end of the experiment, if the subjects’ target emotions were not elicited or if the elicited emotions were insufficiently strong, the data were discarded.

## 3. Methods

### 3.1. EEG feature extraction

(1) Signal preprocessing: The raw EEG data were first processed with a bandpass filter between 0.3–50 Hz (Zheng and Lu, 2015), followed by a 50 Hz trap filter to remove powerline interference. Subsequently, EEG data were downsampled to a 200 Hz sampling rate from 1,000 Hz to reduce the computational complexity and improve the processing speed. To avoid interference from eye-movement artifacts and EMG signals, independent component analysis (ICA) of EEGLAB toolbox (Delorme and Makeig, 2004) was used. Finally, only EEG data from video viewing phase were selected for further processing, and the data from each channel were divided into non-overlapping 1-s samples (Zheng and Lu, 2015; Song et al., 2019) from which EEG features were extracted.

(2) Differential entropy: For EEG signals, differential entropy (DE) is a very effective feature, which is a generalized form of Shannon information entropy over continuous variables (Duan et al., 2013; Zheng and Lu, 2015). Assuming that x is a continuous random variable and f(x) is its probability density function, the differential entropy is calculated by the formula depicted in Eq. 1:

(1) $$h\left(x\right)=-\underset{X}{\int }f\left(x\right)\log\left(f\left(x\right)\right)dx$$

When random variable x ~ N (μ, σ2), Eq. 1 can be rewritten as Eq. 2:

(2) $$\begin{array}{l} h\left(x\right)=-\overset{\infty }{\underset{\infty }{\int }}\frac{1}{\sqrt{2\pi \sigma^{2}}}e^{-\frac{{\left(x-\mu \right)}^{2}}{2\sigma^{2}}}\log\left(\frac{1}{\sqrt{2\pi \sigma^{2}}}e^{-\frac{{\left(x-\mu \right)}^{2}}{2\sigma^{2}}}\right)dx\\ =\frac{1}{2}\log\left(2\pi e\sigma^{2}\right) \end{array}$$

### 3.2. ECG feature extraction

1. Signal preprocessing: Initially, the original ECG signal underwent band-pass filtering using a Butterworth band-pass filter, with a range of 0.4 to 10 Hz. This filtering process aimed to eliminate direct current noise, as well as high-frequency myoelectricity and other interferences. Subsequently, to simplify subsequent signal processing and enhance processing speed, the filtered signal was downsampled. The original sampling frequency of 1,000 Hz was reduced to 200 Hz. Finally, the corresponding video clip of the ECG signal was segmented, and the data from each channel was divided into 60-s segments with a 55-s overlap (Moeyersons et al., 2017; Benouis et al., 2021).

2. Heart rate variability (HRV) feature extraction: Among the various features extracted from the ECG signal, HRV is the most commonly used. It captures the variation in the duration of consecutive heartbeats, as determined by the adjacent R-R intervals. HRV encompasses time-domain, frequency-domain, and some nonlinear features. ECG feature extraction were using TEAP Toolbox (Soleymani et al., 2017). In the time domain, key HRV measures include the Standard Deviation of NN Intervals (SDNN), Root Mean Square Successive Differences of adjacent RR interval lengths (RMSSD), Differences of adjacent RR interval lengths (SDSD), the standard deviation of Successive Differences (SDSD), the number of adjacent RR interval lengths greater than 50 ms (NN50), and the ratio of NN50 to the total number of RR intervals (pNN50). Eqs 3–5 depict the formulas for calculating SDNN, RMSSD, and SDSD, respectively.

(3) $$SDNN=\sqrt{\frac{1}{N}\overset{N}{\underset{i=1}{\sum }}{\left(RR_{i}-\bar{RR_{i}}\right)}^{2}}$$

(4) $$\begin{array}{c} RMSSD=\sqrt{\frac{1}{N-1}\overset{N-1}{\underset{i=1}{\sum }}{\left(RR_{i+1}-RR_{i}\right)}^{2}} \end{array}$$

(5) $$\begin{array}{c} SDSD=\sqrt{\frac{1}{N-1}\overset{N-1}{\underset{i=1}{\sum }}{\left[\left(RR_{i+1}-RR_{i}\right)-\bar{\left(RR_{i+1}-RR_{i}\right)}\right]}^{2}} \end{array}$$

In the equations, N represents the total number of RR intervals, $RR_{i}\;$ represents the length of the ith RR interval, and $\;\bar{RR_{i}}\;$ represents the average length of all RR intervals.

Additionally, the time-domain features encompass geometric characteristics derived from the RR histogram model. These include the HRV Triangular Index (TRI) and the Triangular Interpolation of NN Interval Histogram (TINN) width. The TRI denotes the ratio of the total number of RR intervals to the height of the RR histogram, while the TINN represents the base width of the triangle obtained when approximating the RR histogram with a triangle.

Frequency-domain features are then extracted, primarily including the power of Heart Rate Variability (HRV) in the Very Low Frequency (VLF) range (≤0.04 Hz), Low Frequency (LF) range (0.04–0.15 Hz), and High Frequency (HF) range (0.15–0.4 Hz). Additionally, these features consist of the LF/HF ratio, which represents the ratio of power between LF and HF, as well as the percentage of power contributed by LF and HF components.

Detrended Fluctuation Analysis (DFA) is a quantitative method for analyzing the fractal scaling properties of RR intervals. It is primarily used to measure the short-term and long-term correlations in RR interval sequences. It includes two features: the short-term scaling exponent alpha1 and the long-term scaling exponent alpha2. The calculation steps are as follows:

For a sequence x(i),i=1,2,…,N first calculate its cumulative time series y(k) , as shown in Eq. 6:

(6) $$\begin{array}{c} y\left(k\right)=\overset{k}{\underset{i=1}{\sum }}\left(x\left(i\right)-\bar{x}\right) \end{array}\;\;$$

where $\bar{x}$ is the average of x(i) . Then the obtained time series is divided into $N_{s}$ equally spaced small segments, each of length s , called scale indicators, when s is small, called short-range scale, and when s is large, called long-range scale. A linear trend $y_{s}\left(k\right)$ is fitted to each small segment using the least squares method, and the trend is removed from y(k) to obtain the series after detrending as shown in Eq. 7:

(7) $$\begin{array}{c} \Delta y_{s}\left(k\right)=y\left(k\right)-y_{s}\left(k\right) \end{array}\;$$

Then the volatility function after detrending is shown in Eq. 8:

(8) $$\begin{array}{c} F\left(s\right)=\sqrt{\frac{1}{N}\overset{N}{\underset{k=1}{\sum }}{\left(\Delta y_{s}\left(k\right)\right)}^{2}} \end{array}$$

Finally, the least squares method is used to linearly fit (logs,logF(s)) to these scattered points, and a threshold is set for s. A straight line is fitted when s is less than the threshold, and the slope of the line is the short-range scalar index alpha1, and another straight line is fitted when s is greater than the threshold, and the slope of the line is the long-range scalar index alpha2.

The magnitude of the approximate entropy reflects the complexity of the signal, and the larger the value indicates the higher the complexity of the signal. The calculation steps are as follows: for the sequence x(i),i=1,2,…,N , given the pattern dimension m, a set of m-dimensional vectors can be constructed, as shown in Eq. 9:

(9) X(i)=[x(i),x(i+1),…,x(i+m−1)],i=1,2,…,N−m+1

Set an approximate threshold value r. For any X(i) and X(j) , if the difference between corresponding elements in the two vectors is consistently smaller than r, we say that vectors X(i) and X(j) ,are approximately close under r. Then, for each X(i) , we calculate the ratio of the approximate vectors to the total number of vectors, denoted as $C_{i}^{m}\left(r\right)$ , with the definition in Eq. 10:

(10) $$\varphi^{m}\left(r\right)=\frac{1}{N-m+1}\overset{N-m+1}{\underset{i=1}{\sum }}\ln C_{i}^{m}\left(r\right)$$

Then the approximate entropy is shown in Eq. 11:

(11) $$APEN=\varphi^{m}\left(r\right)-\varphi^{m+1}\left(r\right)\;\;$$

The calculation of approximate entropy involves two parameters, m and r. m is usually taken as 2 and r is usually taken as 0.1 to 0.25 times the variance of the signal.

So far, six time-domain indicators, six frequency-domain indicators and six nonlinear indicators of heart rate variability were extracted. ECG data were analyzed to HRV and heart rate (HR), so a total of 19 ECG features were extracted.

### 3.3. Classification protocol

A linear support vector machine (SVM) was used as a classifier (Chang and Lin, 2011). A block cross-validation method was used to evaluate the classification performance, with data from one block as the test set and others as the training set. This process was repeated five times to ensure that each block was used as the test set. Finally, cross-average classification accuracy was used to evaluate the classification performance.

### 3.4. Comparisons of evaluation results

The EEG in the continuous dimensional emotion model was analyzed by drawing volcano plots referring to (Kane et al., 2022; Mahendran and PM, 2022). Taking valence as an example, all sample features of each subject were first divided into positive and negative groups according to their valence scores. Next, the mean values of all samples with the same valence degree were calculated. Subsequently, a 300-dimensional positive value vector and a 300-dimensional negative value vector for a single subject were obtained. By repeating this process, 15 subjects could form a positive and a negative emotion matrix with a size of 15 × 300, respectively. Paired *t*-tests (Lillietest was conducted, *t* = 0, *p*<0.05) were used to measure the significance of differences between positive and negative emotions in each dimension. The 15 subjects’ average positive emotions and average negative emotions are indicated by the folded line with F. Obviously, F represents the upregulation multiplier of this feature, which was upregulated under a positive emotion rather than a negative emotion. In $\log_{2}\left(F\right)>0$ , this feature is upregulated under a positive rather than negative emotion, otherwise, these features were downregulated. Finally, the above results were plotted as volcano plots. Its X-axis is $\log_{2}\left(F\right)$ , and its Y-axis is $-\log_{10}\left(P\right)$ . The same process was repeated for arousal dimension. Downregulated. Finally, the above results were plotted as volcano plots. Its X-axis is $\log_{2}\left(F\right)$ , and its Y-axis is $-\log_{10}\left(P\right)$ . The same process was repeated for arousal. Dimension.

## 4. Results

### 4.1. Classification results

**Figure 5:**
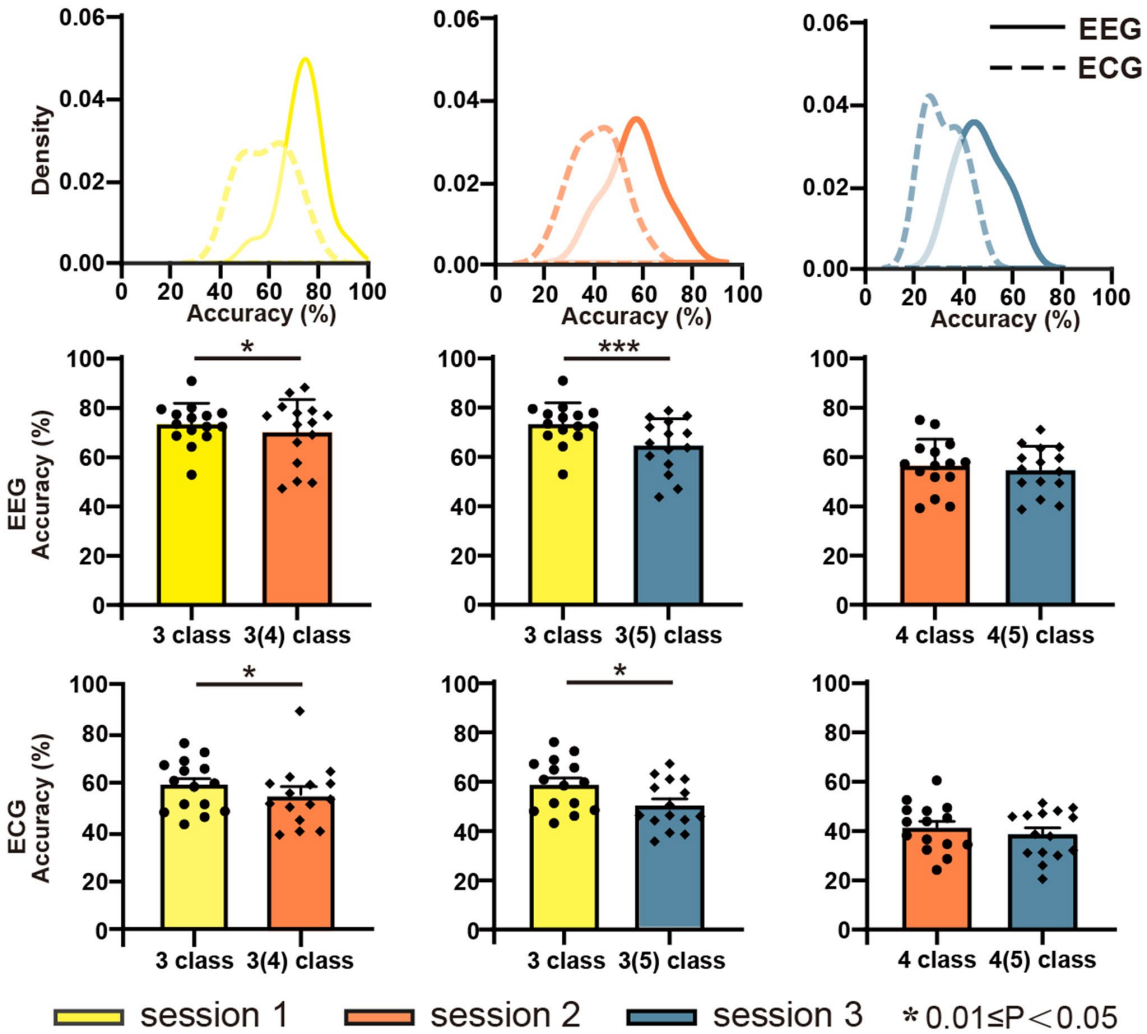
Accuracy of classification recognition of EEG and ECG signals.

**Figure 6:**
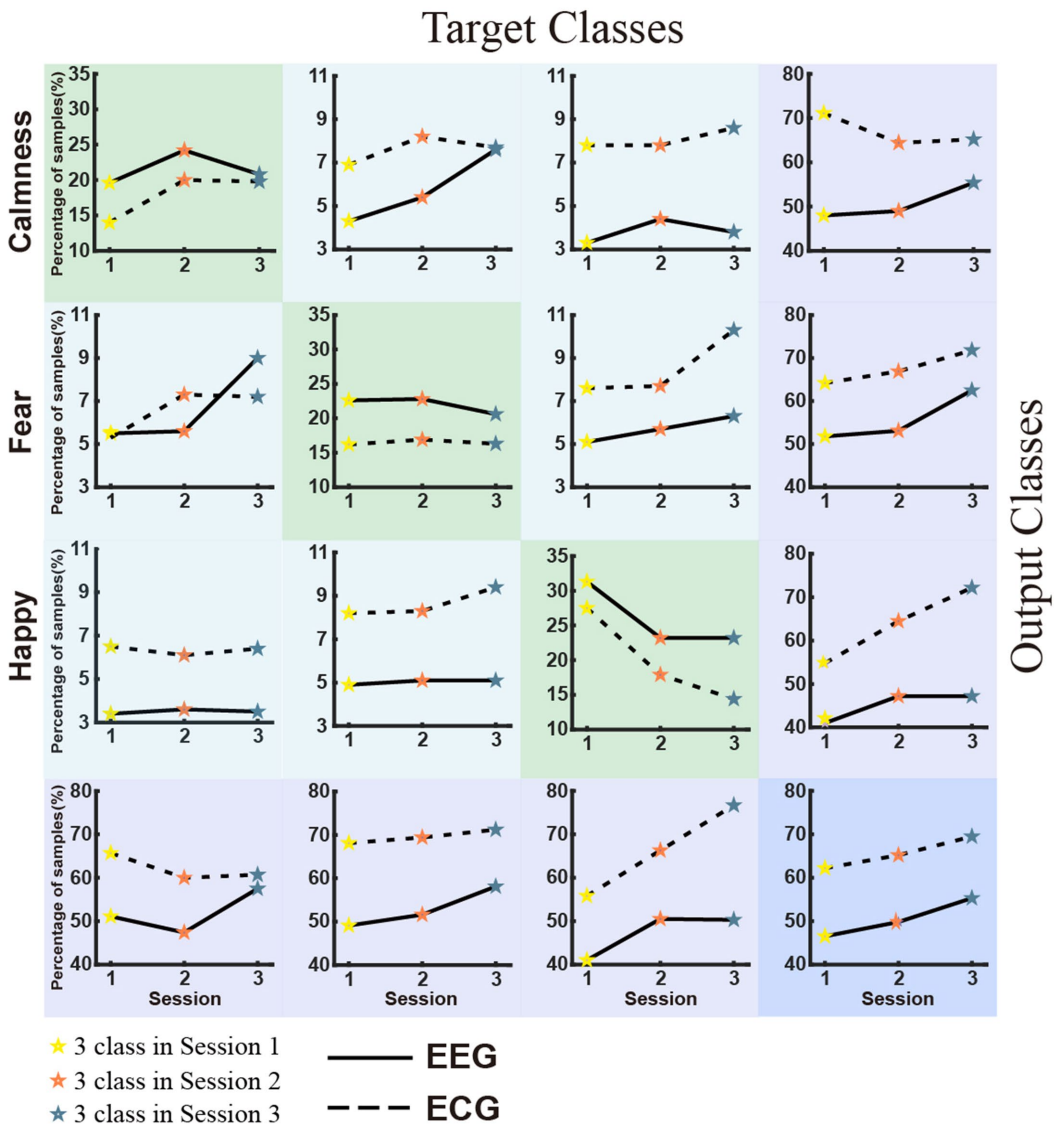
Confusion matrix of the discrete emotion classification experiment.

Figure 5 displays the classification accuracy of discrete emotions. The first row shows the density distributions of the accuracy for sessions 1, 2, and 3 from left to right. Overall, the average classification accuracy decreased as the number of negative emotion types increased. The classification accuracies were 73.50, 56.54, and 46.48% for EEG, and 58.85, 41.42, and 31.47% for ECG in sessions 1, 2, and 3, respectively. In the same session, the average classification accuracy of EEG was significantly higher than that of ECG. Considering the balance of positive and negative emotions, the same emotion types with session 1 were extracted from sessions 2 and 3 and further implemented in the classification experiments. The results are presented in the second row (EEG) and third row (ECG) of Figure 5 Separate comparison experiments passed the independent samples t-test, indicating that the presence of sadness and anger reduced the classification accuracy of happiness, fear, and calmness. Figure 6 depicts the confusion matrix for the discrete sentiment classification experiment. In the off-diagonal figures, we observe that the curves exhibit an upward trend. This suggests that increased from negative emotions increased the error rate of the classification experiments.

### 4.2. Cross-session analysis

**Figure 7:**
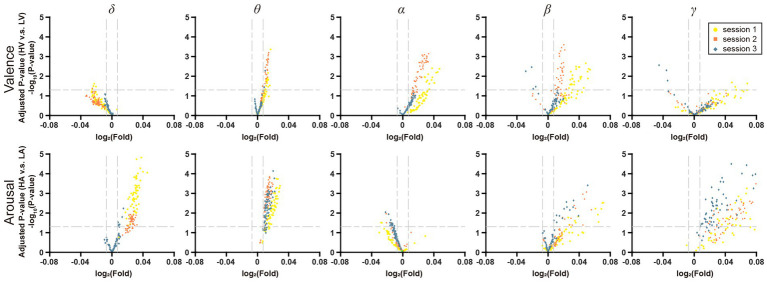
Volcano plot of EEG frequency bands features on VA dimension. The horizontal line in it represents the significant level α = 0.05, and the two vertical lines represent *F* = 201/200 and *F* = 200/201, respectively.

To investigate the effect of increasing the stress of negative emotion, the DE features from five frequency bands (δ, θ, α, β, and γ) were plotted on volcano plots (Figure 7). In terms of valence, there were more points reaching the significant level (Kruskal-Wallis Test, *p* < 0.05) in the θ, α, and β frequency bands and most of them were upregulated, which suggested that these three bands were more critical for distinguishing the valence. In terms of arousal, there were more significant differences in the δ, θ, and β bands, where most points were also upregulated. With increased negative emotional stress, the points in Figure 7 tended to shrink to the original point (0, 0), meaning that the distinguishing ability of these features is vanishing. This trend was more obvious in the θ, α, and β frequency bands for valence and in the δ, θ, and β frequency bands for arousal.

### 4.3. Within-session analysis

**Figure 8:**
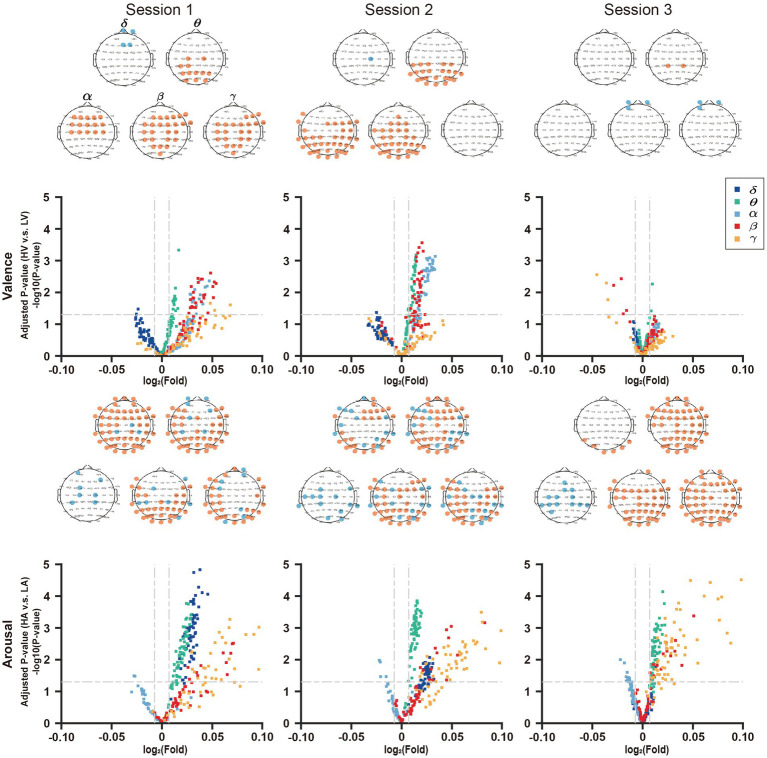
Joint analysis of EEG features in volcano plot and topographic map. In the volcano plots, the horizontal lines in it represents the significant level α = 0.05, and the two vertical lines represent F = 201/200 and F = 200/201, respectively.

To reflect the sensitivity of DE features for valence and arousal in a single session, we mapped the upregulated and downregulated points into the brain map in red and blue, respectively, as shown in Figure 8.

In session 1, the number of features reaching the significant level (*p* < 0.05) in the valence volcano plot were fewer in δ-band than in other bands. Except for the δ-band where four EEG features are painted in blue, the features in other bands are all painted in red, and the number of feature points is more than that of the δ-band. This demonstrated that the other four frequency bands were more sensitive to the changes in valence. In the volcano plots of arousal, there were a certain number of feature points reaching the significant level (*p* < 0.05) in all frequency bands, and most of them were upregulated in the δ, θ, β, and γ bands.

On one hand, regarding the brain map of valence, the prefrontal lobe was downregulated in the δ band. The parietal lobe in the β frequency bands and occipital lobe in the α and θ frequency bands were upregulated to valence’s changes.

On the other hand, for the brain map of arousal, except that parietal lobe in the α band is downregulated, everywhere else of the whole brain is almost upregulated. There were similar results in session 2 and session 3.

In addition, the downregulated region of valence and the upregulated region of arousal in δ-band gradually shrank as the negative emotional stress increased. In contrast, for the arousal, the downregulated region in α-band, upregulated region in γ-band, and downregulated region in θ-band tended to expand from session 1 to session 3.

## 5. Discussion and conclusion

This study analyzed the cause and effect of increasing negative emotions on emotion classification during experimental emotion induction. Several studies have revealed low classification accuracy for negative emotion recognition. For example, in a study by Zhuang et al. negative emotions were identified with nearly 20% lower accuracy than positive and neutral emotions (Zheng et al., 2018; Zhuang et al., 2018). One possible reason was that negative emotions shared some commonalities (Zhuang et al., 2018). However, most of the negative stimulus video clips in the database differed significantly from each other’s dimensional models (at least one dimension) concerning the self-assessment questionnaire. Therefore, we proposed that the difficulty in classifying negative emotions lay not just in the similarity between negative emotions. This coincided with our previous conclusion. Further analysis supported our idea. When negative emotions increased, the physiological activities (EEG and ECG) evoked by the same stimulus material were more similar in characteristics, which made emotion recognition more difficult.

In this study, the volcano plot was introduced to illustrate the DE difference along valence and arousal in different sessions and frequency bands. The vertical axis of the volcano plot indicates value of *p* of one index between two sets of experiment which in our research represent emotional levels (high/low). The horizontal axis of the volcano plot shows the ratio of one index between two sets of experiment. Larger inter-group variations would skew the sample toward the extreme left and right sides of the volcano map. To the best of our knowledge, this is the first application of the volcano plot in the field of emotion recognition. Generally, volcanic plots are used for transcriptome studies as well as genome, proteome, metabolome and other statistical data because they are suitable for showing comparisons between two groups of samples (Edwards et al., 2015; Topper et al., 2017). Obviously, volcano plots provide an effective means for visualizing the direction, magnitude, and significance of changes in samples. Volcano plots like the one shown in our experiment are useful when there are many of observations with a wide range of differences, both positive and negative. Our results show that the volcano plot can efficiently represent the differences of hundreds of DE features (multi-band and multi-channel EEG) induced by several types of emotional materials, which could be worth to popularizing in the field of emotion recognition.

Human emotional states are complex and constantly changing, and there may be similarities and overlaps between different emotional states (Tekoppele et al., 2023). Facing the challenges presented by the indistinct boundaries among negative emotions, firstly we can try to build emotion models with stronger emotion characterization ability to more accurately describe and differentiate different emotional states. Secondly, we can develop more high-quality multimodal databases, such as multimodal physiological signals of text, speech, facial expression, EEG, ECG, respiration. The third approach is to enhance data processing methods, such as adjusting the time window of the EEG signal samples (Shin et al., 2018; Zheng et al., 2018).

Moreover, we believe that imbalanced negative emotional material in the stimulus paradigm can lead to a bias in emotion recognition and affect the accuracy of emotion classification. Considering that this conclusion is true for both EEG and ECG signals, we consider this conclusion to be cross-modal stability. However, the study still has some restrictions. For example, it is unclear whether changing the order of emotional stimulation videos or increasing the types of positive emotion videos will help increase their effect on the excessive negative emotions toward the subjects. Moreover, while more negative emotional stimuli brought more negative emotional experiences for the participants, there was still a lack of a quantitative assessment of the painful experience of watching many negative emotional videos. In addition, the interpersonal response index (IRI) can be considered an indicator to quantify a subject’s painful experience (Lang et al., 2005). Therefore, we plan to introduce more positive emotional stimulus samples in the following study to balance the pain of negative emotional experiences.

## Data availability statement

The original contributions presented in the study are included in the article/supplementary material, further inquiries can be directed to the corresponding author.

## Ethics statement

The studies involving humans were approved by Ethics Committee of Tianjin University (TJUE-2021-138). The studies were conducted in accordance with the local legislation and institutional requirements. The participants provided their written informed consent to participate in this study.

## Author contributions

ZGL, LX, and EWY designed the research study. XMW and SKZ performed the research. SKZ and YY provided help and advice on the experimental paradigm design. XMW and YP analyzed the data. All authors contributed to editorial changes in the manuscript. All authors read and approved the final manuscript. All authors have participated sufficiently in the work and agreed to be accountable for all aspects of the work.

## Footnotes

^1^ https://www.neuroscan.com

^2^ https://www.biopac.com
